# Using a scenario approach to assess for the current and future demand of immunoglobulins: An interview and literature study from The Netherlands

**DOI:** 10.1111/tme.12889

**Published:** 2022-06-24

**Authors:** Praiseldy Langi Sasongko, Marian van Kraaij, Cynthia So‐Osman

**Affiliations:** ^1^ Department of Donor Medicine Research Units Transfusion Technology Assessment and Donor Studies, Sanquin Research Amsterdam The Netherlands; ^2^ Amsterdam UMC University of Amsterdam Amsterdam The Netherlands; ^3^ Department of Home Care Sensire Varsseveld The Netherlands; ^4^ Department of Unit Transfusion Medicine Sanquin Blood Bank Amsterdam The Netherlands; ^5^ Department of Haematology Erasmus Medical Center Rotterdam The Netherlands

**Keywords:** immunoglobulins, IVIG, scenario approach

## Abstract

**Objectives:**

To explore the current and future demand of immunoglobulins globally and specifically for the Netherlands by assessing: (I) which specialties contribute to current demand, (II) new areas of medical need, (III) which transformational factors may impact demand and to what effect, by using a scenario approach.

**Background:**

As immunoglobulin demand continues to increase globally, there is concern of increasing shortages and questions of whether and how future demand will continue based on medical need.

**Methods/Materials:**

In line with scenario principles, a scoping review of Pubmed, Web of Science, Embase and Cochrane and grey literature was conducted. Semi‐structured interviews with subject matter experts were held. The results of the review and interviews were analysed for major themes.

**Results:**

The scoping review resulted in 97 articles, 74 regarding clinical uses, and 23 regarding organisational and other themes. Fifteen clinical and non‐clinical experts were interviewed. I) Neurology, immunology, and haematology were specialties that contribute most to current demand. II) Regarding potential new areas of medical need, the literature review resulted in more indications than the interviews, for example, post‐renal transplants. III) Four groups of key transformational factors were found: factors that could increase immunoglobulin demand (e.g., EMA revisions), decrease demand (e.g., replacement products, Dutch Transfer Act 2021), factors that remain to be seen how it impacts demand (e.g., further evidence), and miscellaneous factors (e.g., supply‐related).

**Conclusion:**

Having identified the specialties and relevant transformational factors that affect immunoglobulin demand, more research is needed on what clinical or organisational strategies would be effective in controlling demand in general for the Netherlands and abroad. Other blood establishments may also use a scenario approach to increase preparedness for future (un)expected developments.

**Abbreviations**
CIDP: chronic inflammatory demyelinating polyneuropathy
EMA: European Medicines Agency
fSCIg: facilitated subcutaneous immunoglobulins
GBS: Guillain‐Barré syndrome
HLA: human leukocyte antigen
IdeS: immunoglobulin degrading enzyme of Streptococcus pyogenes
Ig: immunoglobulins
ITP: immune thrombocytopenic purpura
IVIGs: intravenous immunoglobulins
MG: myasthenia gravis
MMN: multifocal motor neuropathy
NAITP: neonatal alloimmune thrombocytopenia
PDMPs: plasma‐derived medicinal products
PfF: plasma for fractionation
PID: primary immune deficiencies
PSAF: proven specific antibody failure
SC: subcutaneous
SID: secondary immune deficiencies
SmPC: summary of product characteristics

## 1 INTRODUCTION

Since the early 2000s, intravenous immunoglobulins (IVIGs), pooled from the plasma of several thousand individuals, has been a ‘driver’ in determining the demand for plasma for fractionation (PfF) based on medical needs.^1^ IVIG has become prominent for its efficacious ability to treat primary and secondary immune deficiencies and be an immunomodulatory agent for various other disorders.^2^, ^3^, ^4^ Over time, uses for IVIGs have increased as it is used for both registered (on‐label) treatment and non‐registered (off‐label) treatments. Furthermore, administration of immunoglobulins (Igs) has expanded to subcutaneous (SCIg) and facilitated subcutaneous options (fSCIg).^5^, ^6^, ^7^ Thus, the global demand of Igs has increased dramatically over time, particularly in high‐income countries.^8^ A report from MarketsandMarkets™ forecasted that the demand for Ig therapy increased approximately 8% globally and approximately 6% for Europe from 2016 to 2021.^9^ This growing demand leads to increasing costs and supply shortages, which are already occurring in the current system.^2^, ^10^, ^11^ Furthermore, in 2018, Europe obtained approximately 36% of PfF sourced from the United States,^12^ posing challenges for European self‐sufficiency and continuity for the availability of these medicines.

Research from the Marketing Research Bureau found that in 2018, the Netherlands was the 11th highest consuming country of Ig usage per capita.^13^ The Netherlands has experienced decreasing demand for erythrocyte concentrates^14^ which has resulted in fewer recovered plasma (plasma recovered from whole blood collection) and insufficient source plasma (plasma from apheresis). Therefore, with the historical and expected forecast of increased demand for Ig for the Netherlands,^9^ Sanquin, the Dutch national blood bank, needed to make decisions regarding its strategy for the future collection of plasma by assessing the future demand of Ig.

Because Ig demand is a complex topic with various factors and stakeholders, quantitative predictions alone are insufficient and requires understanding of the current and future medical need. Therefore, we employed a scenario approach (also called scenario development or scenario planning) which has been used in other fields such as military, oil and transportation. It seeks to explore multiple plausible futures resulting from trends or policies and aids in long‐term decision making to increase preparedness and proactivity for (un)expected future developments.^15^ This is done by including expert perspectives and systematically identifying relevant transformational factors (broadly defined as developments in society, technology, economy, ecology or politico‐legal) that, in this case, may impact future Ig demand and to what effect (e.g., increase or decrease it).^15^, ^16^ To the best of our knowledge, no study exists that includes qualitative methods in assessing demand, apart from commercialised reports and a study by Health Canada that addresses Ig supply and demand for Canada.^8^ Therefore, the aim of this study is to gain insights into the current and future demand of Ig based on medical need for the Dutch setting. We did this by creating the following research questions:

- Which clinical specialties contribute to the current demand for Ig?
- What are potential new areas of medical need that could be explored?
- What are key transformational factors (from social, technological/clinical, economical, ecological, political, and legal) that would impact Ig demand and to what effect?

## 2 MATERIALS AND METHODS

### 2.1 Setting

In the Netherlands, Ig is distributed in two main ways with a separate reimbursement scheme for each: (1) Intramurally (within hospitals), where Ig for approved indications is reimbursed by insurance with an ‘add‐on’ while non‐approved indications come out of the hospital budget; (2) Extramurally (community pharmacies) where Ig is completely reimbursed by health insurance irrespective of the indication.^17^ Ig is reimbursed for on‐label usage and for certain off‐label indications, which are based on several guidelines and clinicians´ input.^18^

Sanquin is the only blood bank in the Netherlands legally tasked to collect the amount of Dutch plasma necessary for self‐sufficiency. Sanquin Plasma Products (SPP) was a pharmaceutical company that was originally part of the Sanquin Blood Supply Foundation. Albeit now separate, SPP (now Prothya Biosolutions) is still connected to Sanquin and continues its role in obtaining Dutch plasma for fractionation in order to manufacture plasma‐derived medicinal products (PDMPs) for the Netherlands.^19^

### 2.2 Methodology

From February to June 2019, we combined a scoping review with semi‐structured interviews. Following scenario principles, this concurrent methodology was considered ideal to answer the research objectives to examine the topic from existing literature and compare it with the perspectives of experts in the field.

**FIGURE 1:**
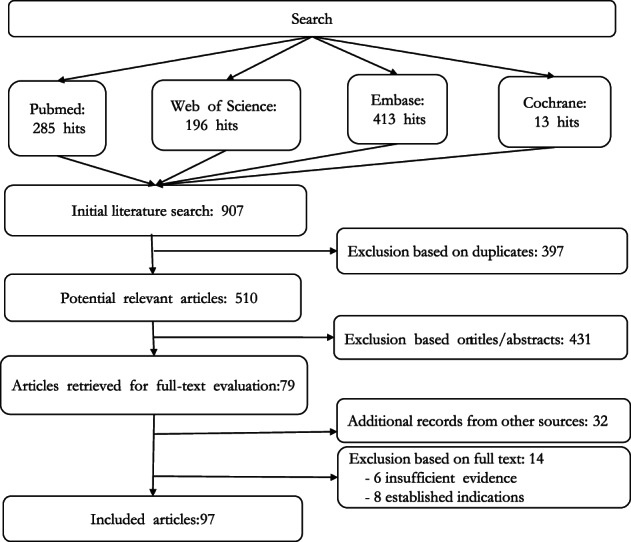
Flowchart of literature search

- *Scoping review*. A scoping review is a type of literature review that provides a broad overview or map of the evidence with expansive inclusion criteria; it may be a precursor to a systematic review.^20^ We created a three‐part search strategy based on the project's aims regarding PDMPs, supply/demand, and current practice, for the time period between January 2010 until July 2019, and then updated from July 2019 to July 2021 (Appendix A). We applied it to several large‐scale databases (Pubmed, Web of Science, Embase, Cochrane). Furthermore, we searched through grey literature (governmental websites, for‐profit and not‐for‐profit plasma websites, presentations from prior conferences). We decided upon three sets of guidelines to be most important for this study: (1) the Australian Criteria (found at https://www.blood.gov.au/igcriteria-version3), which was chosen as the main set of guidelines for referencing other articles to as the ‘Criteria’ is the most recently updated (in 2018) at the time of this study; (2) the revised European Medicines Agency (EMA) guidelines (released January 2019), applicable for the European setting; (3) The Dutch transfusion guidelines (CBO) 2020, applicable for the Netherlands. For all searches, inclusion criteria included articles from January 2010 to July 2021 in the English and Dutch languages. We excluded studies regarding animals, ‘on‐label’ or ‘established’ indications with no changes (e.g., dosage) to it, and conference proceedings and abstracts if full text was not available. Literature was also ‘snowballed,’ meaning that it was obtained through interviews or as a citation from another article. Authors PLS and CSO independently performed the screening of titles, abstracts, and full‐text articles. When needed, discussions were held between both authors in order to reach consensus. After eliminating duplicates, and reading for titles and abstracts, 97 articles were chosen for full text (Figure 1).
- *Semi‐structured interviews*. We used a purposive sampling strategy^21^ to identify experts such as clinicians in specialties known to use (the most) Ig, SPP personnel, and representatives from a patient organisation and a not‐for‐profit plasma association. All experts were initially approached through a standardised email invitation providing information about the study; if they agreed to participate, each interview would last approximately 30–60 min in length and was recorded with informed consent. Experts were assured of anonymity and confidentiality in the publication of their data and gave verbal consent prior to being interviewed. Interviews were in person or over the telephone, with one expert only accessible through email. Additional respondents were ‘snowballed,’ a method in which prior respondents were asked to refer other individuals they thought would be appropriate for this study. An interview guide was created in accordance with the research questions comprising of three main sections: (I) current IVIG usage (for clinicians) or distribution/trends (for non‐clinicians), (II) transformational factors in society, technology/clinical, economics, politics, or legal that could impact IVIG demand and to what effect, (III) future prediction of IVIG demand (Appendix B). A semi‐structured approach was adopted so that the interview guide was followed with additional probing and follow‐up questions when appropriate. This allowed for the interviews to be more conversational in nature.^22^ Interviews were deemed sufficient in accordance with the project's timeline and the scoping literature review findings.

*Analysis of interviews*. All recorded interviews were transcribed verbatim and coded using qualitative software MAXQDA 2018 (VERBI Software GmbH, Germany). Several rounds of coding were applied to understand the data in their context. First cycle coding followed a pre‐determined coding framework based on the research aims.^23^ Author PLS and two other researchers assessed three transcripts initially to review and revise the coding framework. Coding differences were settled through consensus and adjustments were made to the framework. The framework was expanded to allow for inductive themes that arose. Author PLS coded the rest of the transcripts with this adjusted framework, and author MvK checked five random transcripts for quality assurance.

## 3 RESULTS

Of the 15 experts interviewed, 8 (53%) were clinician‐researchers representing 6 specialties; almost all experts had 11+ years working in their respective fields (Table 1).

**TABLE 1:** Descriptives of interviewed experts

Expert characteristics	Number of Experts from total (*n* = 15)
Male	10 (67%)
Female	5 (33%)
Occupation	
Clinician‐researcher	8 (53%)
Hospital pharmacist	1 (7%)
Patient organisation representative	1 (7%)
Not‐for‐profit plasma association representative	1 (7%)
Sanquin Plasma Products employees	3 (20%)
Immunoglobulin scientist	1 (7%)
Years of experience	
0–10	1 (7%)
11–20	8 (53%)
21+	6 (40%)
Clinical specialties represented	Neurology, Nephrology, Dermatology, Immunology, Haematology, and Haemato‐oncology

Of the 97 articles obtained from the scoping review, 74 concerned clinical factors, subdivided by specialty, and 23 regarded supply, demand, or organisational actions (Supplementary Table 1).

To answer the research questions, the literature is reviewed (*a*) followed by the interviews (*b*). For *b*, all quotations were directly taken from the experts.

### 3.1 Clinical specialties that contribute to Ig demand

- From both the literature and interviews, it was found that there is no centralised monitoring system that monitors Ig demand across the different specialties in the Netherlands—only national numbers of certain product usage (i.e., Nanogam)^24^ and private, hospital‐based numbers. However, it was assumed that the Dutch setting was similar to other high‐income countries like the UK: the National Database Annual Reports for 2017–2020 reported Ig use by specialty by volume were (in order of) neurology, immunology, and haematology.^25^, ^26^, ^27^
  Further, documents such as the EMA Guidelines and Dutch transfusion guidelines (CBO Consensus 2020) are applicable for the Dutch setting. As of January 2019, the EMA Guidelines have been amended and expanded for the European setting^28^ (Table 2 describes these changes including its impact for multiple specialties). The CBO Consensus 2019 (unchanged from 2011) also specifies Ig to be indicated in pregnancy when fetal and neonatal alloimmune thrombocytopenia (FNAIT) is suspected,^35^ which is also an established therapeutic role in the Australian Criteria^36^ and literature.^27^, ^37^
- Clinicians could provide examples of specific specialties in which demand has grown, including internal medicine (specifically oncology, humoral immune deficiencies and haematology). Furthermore, 6 experts described the increased demand in neurology, where the amount of CIDP and MMN patients are fewer but have higher consumption: *‘When it comes to number of patients, those with [primary] immune deficiencies is a high number. But if you look into usage of grams or kilograms of products, then the neuromuscular disorders (the MMN or CIDP) consume more. There are less patients, but the dosage is much higher’. (Expert 5)* Moreover, the growth in neurology underlies another significant growth in the area of secondary immune deficiencies (SIDs). Five experts had noticed this trend with similar explanations surrounding patient numbers (‘*for 1 PID patient, you have 20 or 30 SIDs’ (Expert 5)*) and Ig's efficacy in treating SIDs (‘*we learned from experience that Ig really works not only in PIDs but also in SIDs’ (Expert 14)*.

**TABLE 2:** Description of European Medicines Agency Guideline revisions and the specialties affected

Indication type	Definition	Specialties affected
I) Replacement therapy in adults, and children and adolescents age 0–18 years	For replacement therapy, IVIG should be initiated in Primary immunodeficiency syndromes (PID) with impaired antibody production and Secondary immunodeficiencies (SID) in patients who have proven either specific antibody failure (PSAF) or serum IgG level of <4 g/L *and* suffer from severe or recurrent infections, ineffective antimicrobial treatment**	Immunology, oncology, haematology, haemato‐oncology, paediatrics
II) Immunomodulation in adults, and children and adolescents age 0–18 years.	For immunomodulation, IVIG is indicated in five specific diseases in which they need maintenance dosages for longer periods of time due to the chronic nature of the diseases. Primary immune thrombocytopenia (ITP), in patients at high risk of bleeding or prior to surgery to correct the platelet count (including ITP in pregnancy^29^) Guillain Barré syndrome (GBS) Kawasaki disease (in conjunction with acetylsalicylic acid) Chronic inflammatory demyelinating polyradiculoneuropathy (CIDP)^30^, ^31^, ^32^, ^33^ ^a^ Multifocal motor neuropathy (MMN)^33^, ^34^ ^a^	Haematology, neurology, paediatrics

^a^ Newly‐added indications as part of the 2019 revisions.

### 3.2 Potential new areas of medical need for immunoglobulins

With regards to potential new areas of medical need and new indications for Ig, the literature search provided more indications than the interviews.

- The literature provided a number of possible Ig treatment options in infectious diseases such as encephalitis,^38^, ^39^ in solid organ transplant patients,^40^, ^41^ in dermatology such as atopic dermatitis,^42^, ^43^, ^44^, ^45^ in immunology and rheumatology, such as systemic lupus erythematosus,^46^, ^47^, ^48^ to treat sepsis,^49^, ^50^, ^51^ and in women with reproductive failure.^52^, ^53^
- Nine of the 15 experts gave suggestions on new areas of medical need, although only a few (3/9) could provide specifics: in dermatology, soft tissue infections; in neurology, small fibre neuropathy or myositis; in infectious diseases, Ebola or dengue (with convalescent plasma). The other experts provided generalised ideas for ‘new indications’ in the realms of aging‐related, autoimmune, and/or immune‐modulating diseases, and other SIDs.

### 3.3 Key transformational factors that could impact immunoglobulin demand and its subsequent effect

When literature was searched and experts were asked regarding transformational factors that could impact Ig demand and to what effect, three groups emerged: factors that could increase Ig demand, factors that could decrease Ig demand, and factors that remain to be seen how it impacts demand. (Supplementary Tables 2–4 list these factors in‐depth and by which method they were found ‐ scoping review or interview).

#### 3.3.1 Factors that could increase demand

**FIGURE 2:**
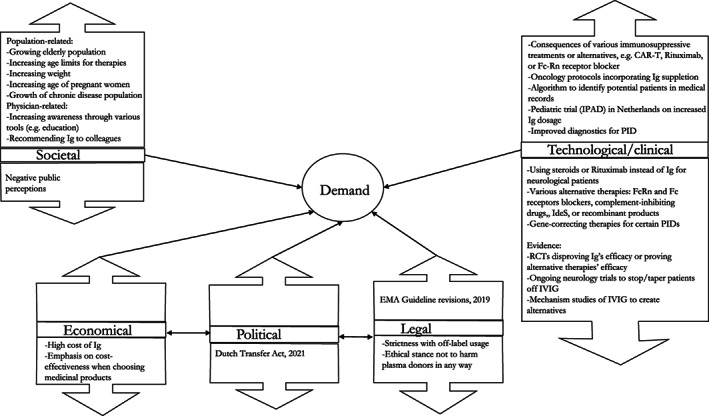
Relevant transformational factors that could increase (“up” arrows) or decrease (“down” arrows) demand

Both the literature review and interviews revealed social, technological, economic, political, and legal factors that could increase demand (shown in the ‘up’ arrows in Figure 2). With regards to social factors, both methods described demographic factors, such as age (the growing elderly patient population,^6^, ^40^, ^51^, ^54^ the older age of women becoming pregnant^52^ and heightened age limits for various therapies) and increasing weight (as treatment is weight‐based).^51^, ^54^ From a communication perspective, experts shared that increasing physician awareness (through education, diagnostic tools) and interactions (word‐of‐mouth) may also contribute to increasing demand.^51^ Two clinician‐researchers cautioned that while these factors may increase demand, this may not be entirely applicable to paediatrics due to the small number of paediatric patients with PIDs, whereas another clinician‐researcher stated that as more adults are being diagnosed with PIDs, there would be heightened demand due to the chronic and weight‐based natures of treatment.

Linking both social and technological/clinical factors, experts stated that as increasing age is no longer a limiting factor for treatments, and as new cellular and immunosuppressant therapies and drugs are developed, patients endure a prolonged immunosuppressive state that possibly warrant Ig as secondary support.^6^, ^51^, ^55^, ^56^, ^57^, ^58^, ^59^ Lastly, one expert familiar with the EMA Guideline revisions stated it could justify increased Ig demand in Europe, particularly for SIDs.

#### 3.3.2 Factors that could decrease demand

Both the literature review and interviews revealed technological/clinical, and economic‐political factors that could decrease demand (shown in the ‘down’ arrows in Figure 2). With regards to technological/clinical factors, alternative therapies were found that overlap with Ig's mechanisms of action, such as the neonatal receptor (FcRn)^60^ and other Fc receptor blockers,^61^ complement‐inhibiting drugs,^62^, ^63^ and reduction of antibody production.^64^ A clinical expert spoke of the value of trials seeking to taper/stop neurology patients off of Ig or switching to Rituximab instead (which would impact demand as neurology is the highest consuming specialty). Interestingly, from both interviews and the literature, limitations of these treatments or therapies were found: certain treatments (e.g., FcRn, Rituximab) held a ‘double edge’ in being a possible alternative for patients, and yet also causing immunosuppression (as described previously), which could still warrant Ig, albeit reduced.

Further, while gene‐correcting options are available for PIDs,^65^ it is only suitable for a minority: *‘Only 10 to 15% of patients have a monogenetic cause of the diseases, and that means that only that percentage would be suitable to undergo these procedures. That's one. Second, there's no gene therapy for CVID or XLA’. (Expert 12)* Overall, while experts acknowledged the possibilities of alternative therapies and its impact on decreasing Ig demand, many expressed they see no real competition that would displace Ig within the next 3–10 years.

Additionally, the expense of Ig was an important economic factor with many ramifications^1^, ^66^, ^67^, ^68^, ^69^, ^70^ which could potentially force economies to curb their demand: *If the cost is higher, the consumption can decline because countries cannot afford it (Expert* 3). One clinician particularly tied evidence, economic and legal aspects together by advocating for a strict evidence‐based approach to prescribing Ig, which would dampen demand: ‘*I think we should strive for more evidence‐baseness of using the IVIG, I think the present situation is still a little bit wild and uncontrolled…there will be a stop on that, it will be more strictly regulated…If IVIG would not be reimbursed for specific indications because the evidence is meagre or shallow, then of course, that will influence prescription’. (Expert 10)* Literature provides examples of how some hospitals have initiated various stewardship programs to monitor and curb demand.^59^, ^71^, ^72^

More social, ecological, political, legal factors were found from the interviews. Particular for the Netherlands, experts shared how an important healthcare change was to occur in 2021, called the *overheveling*, or the Transfer Act, motioned by the Ministry of Health in 2017 to curb Ig demand by narrowing Ig prescription and distribution to the hospitals only. This act would eradicate the local pharmacy scheme, a ‘black box’ where reimbursement occurs regardless of the indication. Those patients and their treatment costs would be transferred to the hospital budget instead. Experts hypothesized of the upcoming effects, with some perceiving advantages such as providing insights into patient care and usage, ‘*to give a bigger opportunity to actually cut down on cost’* (*Expert 13*). However, they seemed quite wary of the disadvantages, such as patients being referred from hospital‐to‐hospital or patients having limited product options.

#### 3.3.3 Factors that remain to be seen on how it impacts demand

Both the literature and interviews stated that evidence from more high‐quality RCTs is needed^30^, ^38^, ^44^, ^49^, ^51^, ^73^, ^74^ and would impact demand in the direction of the results (i.e., if the evidence proved that Ig is truly efficacious and/or have increased dosage of the product, then it would potentially increase demand, but if the evidence proved otherwise, then it would decrease demand). Further, if studies could clearly elucidate IVIG's mechanisms of action,^75^ then viable alternatives could be created. Additionally, some experts noted a trend in their patients regarding increased usage of SCIg or fSCIg. A study of Dutch neurologists stated that the effects of the PATH study^30^ could result in increased usage of SCIg amongst CIDP patients.^76^ Further, more studies are being done with fSCIG as the hyaluronidase allows for larger volumes at singular subcutaneous sites and thus, fewer doses.^6^, ^7^, ^77^ Literature states that SCIg is cost‐saving^31^, ^78^, ^79^, ^80^, ^81^ with yet unknown consequence on demand.

#### 3.3.4 Miscellaneous but important factors

**FIGURE 3:**
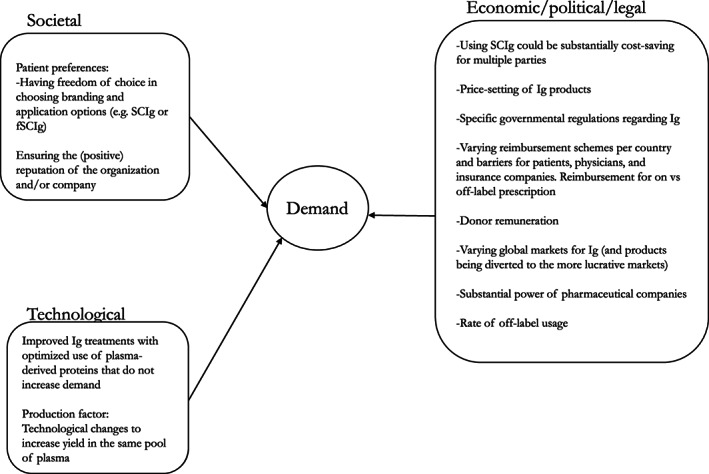
Miscellaneous transformational factors that impact demand

**FIGURE 4:**
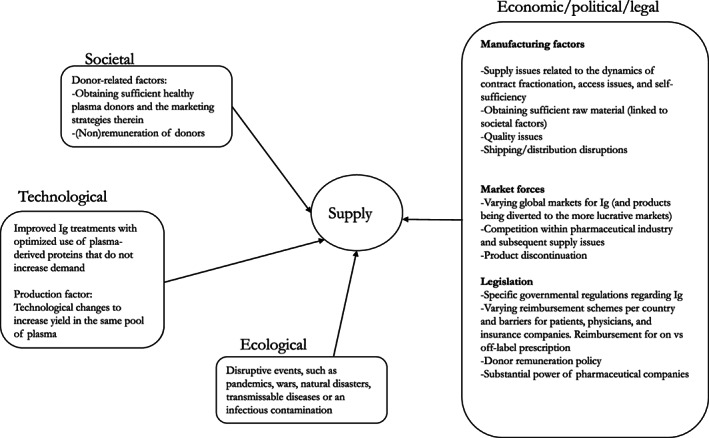
Miscellaneous transformational factors that impact supply

As demand is linked with supply, relevant factors arose that affect both (Figures 3 and 4). These include supply shortages due to the ‘system obstructions’, as coined by one expert, which reflect societal,^82^, ^83^, ^84^ economic/political/legal^25^, ^27^, ^59^, ^85^, ^86^, ^87^, ^88^, ^89^ and ecological factors embedded and interrelated within the dynamics of contract plasma fractionation,^1^ such as market forces, manufacturing issues and infectious contamination^59^ (Figure 4). Overcoming shortages, therefore, require attention to these various systemic elements.

## 4 DISCUSSION

This study sought to gain insights into the future demand of Ig in the Netherlands by using a scenario approach. This led to the identification of relevant groups of transformational factors that could increase, decrease, or impact (with yet unknown effect) the future demand of Ig for the Netherlands, with global implications as well.

Although there is no Dutch centralised monitoring system, current demand lies within the specialties of neurology, immunology, and haematology similar to other countries.^8^, ^25^, ^27^, ^54^ As the latter two are part of 11 sub‐specialties within Internal Medicine, it is conceivable that Internal Medicine and neurology are the main consumers of Ig in the Dutch context. In a study amongst Dutch neurologists treating CIDP, substantial variation in diagnosis and treatment options were found, with authors stating there is potential in decreasing IVIG usage if there was clearer advice in the European Federation of Neurological Societies/Peripheral Nerve Society guidelines.^76^ Healthcare Institute Netherlands has a database that shows how Nanogam's users have increased from 2016–2020 and it is the third most expensive drug to reimburse.^24^ Hence, at the beginning of this study in 2019, the then‐upcoming Transfer Act in 2021 was perceived as a major political change to create transparency and oversight into Ig prescription to reduce demand from unnecessary indications^90^ but it was cancelled indefinitely due to COVID‐19.^91^

What is applicable for both the Netherlands and high‐income countries are the following transformational factors, subdivided into specific factors for immune deficiencies and immunomodulation. With regards to PIDs, there could be increased demand as experts highlighted that clinicians are growing in awareness of diagnosing PIDs and using Ig treatment which may lead to increased demand,^92^ particularly in diagnosing and treating *adult* patients.^93^ A study using latent therapeutic demand modelling showed that the potential demand for treating CVID or XLA (two of the most common PIDs using Ig) actually exceed current demand, meaning that more Ig could be used to treat these conditions.^94^, ^95^ Conversely, gene‐correcting therapies could counterbalance this demand, although it is currently suitable for certain monogenetic causes of disease and is not yet applicable for CVID or XLA.^65^ As SIDs occur as a consequence of disease or therapy,^4^ SID demand could continue increasing with the rise of immunosuppressive therapies, such as CAR‐T cell,^56^, ^57^ compounded with the effects of socio‐demographic factors (increasing age, weight). The effect of the latter could be hampered if there was standardisation of dosing based on ideal or adjusted body weight but wide variations in practice still persist.^96^, ^97^, ^98^ Arguably, Ig need not be the first line of treatment options,^4^, ^99^ but the broadened EMA definition for SIDs^28^ provides allowance for it to be.

Furthermore, we found four transformational factors that impact both immune deficiencies and immunomodulation. First is the EMA 2019 guideline revisions, which gives prescribing justification for reimbursement agencies, physicians, and other stakeholders in Europe. Its inclusion of CIDP and MMN into its approved indications reflect current practice and justifies its continual (and possibly, increased) use. This latter point is reflected in CSL Behring's growth of IVIG and SCIg sales in early 2020 due to the inclusion of CIDP in its product labeling.^100^ However, the impact for possible increase within Europe will vary depending upon country‐specific reimbursement policies. Secondly, the rise of possible alternative therapies, particularly, FcRn receptor blocker^61^, ^101^ and complement inhibiting drugs,^63^ could curb demand significantly if it were successful. Whatever replacement or alternative products or therapies are produced must be proven to be safe and (cost) effective in order to be truly viable options. One example of such is eltrombopag, found to be a noninferior but more cost‐effective option than IVIG as bridging therapy for ITP.^102^ The evidence from trials of these alternatives or basal studies of Ig's mechanisms will contribute to a body of evidence (the third element) that will be tale‐telling to the direction of demand. Lastly, the rising popularity of SCIg^103^ and fSCIg^7^, ^77^ have a questionable impact on demand, although studies show its economic beenfit.^7^, ^31^, ^78^, ^79^, ^80^ Pharmacokinetic and clinical studies suggest that switching from IVIG to SCIg requires a higher dosage,^104^, ^105^ which would result in increased demand,^104^ whereas others found that a 1:1 dosage is comparable and/or equally effective.^30^, ^79^, ^106^ and the FDA recommends a conversion rate of 1.4. Further RCTs are needed to determine the most beneficial or personalised dosing strategy.

Hence, future demand for the Netherlands and other high‐income countries is likely to increase given strong demand patterns,^9^, ^13^ and the aforementioned factors that could increase demand. However, the growth of the demand will also be abated by the individual and cumulative effects of the transformational factors noted above. Thus, for clinicians and policy makers, it is necessary to monitor both aspects in making decisions regarding Ig sufficiency. For the Netherlands, one way would be to create a centralised monitoring system, such as the UK's National Immunoglobulin Database.^27^ Such a system could also monitor the factors mentioned here, include changes to guidelines or in prescribing practices, for the sake of assessing effect and making more accurate predictions for the country's future demand. Furthermore, measures to steward its use are necessary, and a first step would be to conduct local/regional audits regularly. One promising area is further research into clinician awareness and prescribing behaviour, the core activity of Ig demand. This includes better understanding of psychological factors, group dynamics, and even logistical reasons for why various initiatives in hospitals to control demand are not successful for the long‐term.^54^ Additionally, lessons can be learned and replicated from the hospital stewardship programs/organisational interventions that have sprung up, which exhibits varying levels of (dramatic) success in curbing Ig usage.^59^, ^71^, ^72^

Strengths of this study include using the combination of a scoping review and interview study that allowed for identifying the contextual factors related to Ig demand. This methodology is customizable for any country, in that it highlights country‐specific issues regarding the future demand of Ig and the implications thereof. Using this approach could be an alternate, and more independent (non‐biased) approach to purchasing reports from commercial companies. Other blood establishments could use this work as a starting point and modify it accordingly, even taking it further by assessing the mitigating measures to mitigate the threats and embrace the opportunities,^107^ or using traditional scenario methodology and choosing to focus on certain transformational factors to create specific future scenarios.^15^ This methodology was used by Sanquin to make recommendations to the Ministry of Health regarding the need for increased plasma collections, which resulted in the opening of the first plasma‐only collection in the Netherlands center in 2020.

The main limitation of this study was its lack of numerical data for the Dutch setting for questions such as the number of patients requiring Ig, estimates of future demand or extrapolating trends. Further, in interviewing 15 experts, of which 8 are clinicians, with one neurologist and two haematologists, we may have missed other, or more nuanced, perspectives. Due to the limited time frame of this study, we made choices to interview a wide range of key experts and chose these clinicians as representatives of their fields due to their reputation and output, which may have introduced a selection bias. Additionally, including SPP employees may have also introduced bias, but these persons were chosen for their content knowledge and their remarks were compared and validated with clinicians and the other representatives during interviews.

## 5 CONCLUSION

Using a scenario approach, we have identified that neurology, immunology, and haematology are the main drivers of Ig demand along with four groups of transformational factors that may impact demand. Future demand for the Netherlands and other high‐income countries is expected to continue, but may be abated by the individual and cumulative effects of the other factors. Hence, monitoring demand patterns and its contributing factors are needed to facilitate responsible use of Ig along with thinking of various long‐term strategies on the local, clinical, and national levels that will aid in preparing for future Ig developments, whatever they may be.

## AUTHOR CONTRIBUTIONS

MvK conceived the initial idea of the study, and all authors designed the methodology. PLS conducted the interviews and analyses and MvK partially reviewed analysis. PLS and CSO conducted the scoping review and analyses. PLS wrote the manuscript while CSO and MvK critically reviewed and revised the manuscript.

## FUNDING INFORMATION

Funding for this work was supported by Sanquin internal research grant PPOC‐L2245.

## CONFLICT OF INTEREST

No conflicts of interest to disclose.

## ETHICS STATEMENT

This study did not require ethical approval due to its scoping review nature, and lack of personal contact with donors, patients, or vulnerable groups.

## Supporting information


**Supplemental Table 1** List of scoping review results by specialty and/or topic.

**Supplementary Table 2** Factors that could increase Ig demand
**Supplementary Table 3**: Factors that could decrease Ig demand.
**Supplementary Table 4**: Miscellaneous but important factors

## Data Availability

The authors confirm that the data supporting the findings of this study are available within the article and its supplementary materials. Further data concerning the scoping review is available from the corresponding author, CSO, upon reasonable request.
