# Supplementary material for: Using a scenario approach to assess for the current and future demand of immunoglobulins: An interview and literature study from The Netherlands
Source: Transfus Med. 2022 Jun 24;32(5):410–21. doi: 10.1111/tme.12889 (PMC9795925; doi:10.1111/tme.12889)
Supplement: Supplementary file 1 — Supplemental Table 1 List of scoping review results by specialty and/or topic. [file TME-32-410-s002.docx]

**Supplemental Table 1:** List of scoping review results by specialty and/or topic

| **Specialty and/or topic** | **Studies by first author** |
| --- | --- |
| **Dermatology** | Ehsani (2016)^1^  Enk (2016)^2^  Forbat (2018)^3^  Forbat (2018)^4^  Heelan (2014)^5^  Thomas (2020)^6^ |
| **Internal Medicine**  (Includes nephrology, geriatrics, oncology, acute internal medicine, ICU, pharmacology, immunology and allergic diseases, infectious diseases, endocrinology, hematology, transfusion medicine) | Aringer (2012)^7^  Abboud (2021)^8^  Goddard (2020)^9^  Khellaf (2011)^10^  Na (2019)^11^  Nahirniak (2010)^12^  Nierhaus (2020)^13^  Michel (2015)^14^  Reiser (2017)^15^  Soares (2014)^16^  Wasserman (2020)^17^  Wiesik-Szewczy (2020)^18^ |
| **Neurology** | Absoud (2016)^19^  Eftimov (2013)^20^  Furlan (2016)^21^  Gelinas (2019)^22^  Hughes (2008) ^23^  Lazzaro (2014)^24^  Le Masson (2018)^25^  Maheshwari (2018)^26^  Meyer (2018)^27^  O’Connell (2020)^28^  Press (2016)^29^  Rajabally (2014)^30^  Sanders (2018)^31^  Stangel (2016)^32^  Tsai (2010)^33^  van Schaik (2018)^34^  Verboon (2019)^35^  Winters (2011)^36^  Yokoyama (2017)^37^ |
| **Obstetrics** | Sung (2017)^38^  Shaulov (2020)^39^ |
| **Obstetrics and hematology** | Callec (2016)^40^  Okubo (2018)^41^  Wegnelius (2018)^42^ |
| **Obstetrics and nephrology** | Colla (2018)^43^ |
| **Pediatrics** | Barut (2015)^44^  Lieberman (2019)^45^  Prefumo (2019)^46^ |
| **Pediatrics and immunology** | Shehata (2010)^47^  Vultaggio (2015)^48^ |
| **Pediatrics and infectious diseases** | Iro (2017)^49^ |
| **Pediatrics and neurology** | Gadian (2016)^50^  Hopkins (2017)^51^  Nosadini (2016)^52^ |
| **Pediatrics and rheumatology** | Rodriguez (2017)^53^  Barut (2015)^44^ |
| **Surgery and internal medicine** | Florescu (2019)^54^  Shehata (2010)^55^ |
| **Combination**  (more than 2 main specialties combined) | Barmettler (2018)^56^  Bhella (2018)^57^  Brand (2021)^58^  Calizzani (2013)^59^  De Angelis (2013)^60^  Farrugia (2012)^61^  Farrugia (2016)^62^  Grazzini (2013)^63^  Kohn (2017)^64^  Lanzoni (2013)^65^  Lanzoni (2013)^66^  Murphy (2019)^67^  O Mahony (2013)^68^  Perez (2017)^69^  Shapiro (2014)^70^  Strengers (2016)^71^  Strengers (2017)^72^  Watad (2017)^73^  Blau (2016)^74^ |
| **Demand-related** (including the modeling of it) | Grazzini (2013)^75^  Stonebraker (2014)^76^  Stonebraker (2018)^77^  Farrugia (2021)^78^ |
| **Supply-related** (including self-sufficiency, donors and donations, optimization of plasma-derived proteins) | Cheraghali (2010)^79^  Farrugia (2009)^80^  Flanagan (2017)^81^  Godin (2013)^82^  Rautonen (2010)^83^  Spath (2017)^84^  Veldhuizen (2013)^85^  Health Canada (2018)^86^ |
| **Economical/political/legal/ethical factors** related to supply and demand | Ducruet (2013)^87^  Grabowski (2016)^88^  Perraudin (2020)^89^  Petrini (2014)^90^  Prevot (2020)^91^  Shapiro (2012)^92^  Winddeger (2019)^93^ |
| **Organizational actions** (includes stewardship programs) | Derman (2021)^94^  Edington (2020)^95^  Jones and Wilkie (2016)^96^  Tsapepas (2019)^97^ |

References

1. Ehsani-Chimeh N, Marinkovich MP. Practice and Educational Gaps in Blistering Disease. *Dermatol Clin* 2016; **34**(3): 251-6.

2. Enk AH, Hadaschik EN, Eming R, Fierlbeck G. European Guidelines (S1) on the use of high-dose intravenous immunoglobulin in dermatology. 2016.

3. Forbat E, Ali FR, Al-Niaimi F. Intravenous immunoglobulins in dermatology. Part 1: biological mechanisms and methods of administration. *Clin Exp Dermatol* 2018; **43**(5): 513-7.

4. Forbat E, Ali FR, Al-Niaimi F. Intravenous immunoglobulins in dermatology. Part 2: clinical indications and outcomes. *Clin Exp Dermatol* 2018; **43**(6): 659-66.

5. Heelan K, Hassan S, Bannon G, et al. Cost and Resource Use of Pemphigus and Pemphigoid Disorders Pre- and Post-Rituximab. *J Cutan Med Surg* 2015; **19**(3): 274-82.

6. Thomas RM, Colon A, Motaparthi K. Rituximab in autoimmune pemphigoid diseases: Indications, optimized regimens, and practice gaps. *Clin Dermatol* 2020; **38**(3): 384-96.

7. Aringer M, Burkhardt H, Burmester GR, et al. Current state of evidence on 'off-label' therapeutic options for systemic lupus erythematosus, including biological immunosuppressive agents, in Germany, Austria and Switzerland--a consensus report. Lupus; 2012. p. 386-401.

8. Abboud H, Probasco JC, Irani S, et al. Autoimmune encephalitis: proposed best practice recommendations for diagnosis and acute management. *Journal of neurology, neurosurgery, and psychiatry* 2021; **92**(7): 757-68.

9. Goddard S, Hughes D, Diwakar L, Graham J. Impact of stopping long-term immunoglobulin therapy in haematological secondary antibody deficiency. *British Journal of Haematology* 2020; **189 (Supplement 1)**: 91.

10. Khellaf M, Michel M, Quittet P, et al. Romiplostim safety and efficacy for immune thrombocytopenia in clinical practice: 2-year results of 72 adults in a romiplostim compassionate-use program. *Blood* 2011; **118**(16): 4338-45.

11. Na IK, Buckland M, Agostini C, et al. Current clinical practice and challenges in the management of secondary immunodeficiency in hematological malignancies. *Eur J Haematol* 2019.

12. Nahirniak S, Hume HA. Guidelines for the use of immunoglobulin therapy for primary immune deficiency and solid organ transplantation. *Transfus Med Rev* 2010; **24 Suppl 1**: S1-6.

13. Nierhaus A, Berlot G, Kindgen-Milles D, Müller E, Girardis M. Best-practice IgM- and IgA-enriched immunoglobulin use in patients with sepsis. *Annals of intensive care* 2020; **10**(1): 132.

14. Michel M, Adoue D, Cheze S, et al. French observatory of adult' chronic immune thrombocytopenia (ITP) treated by thrombopoietin receptor agonists (TPO-RAS). *Blood* 2015; **Conference**(var.pagings): 2250.

15. Reiser M, Borte M, Huscher D, et al. Management of patients with malignancies and secondary immunodeficiencies treated with immunoglobulins in clinical practice: Long-term data of the SIGNS study. *Eur J Haematol* 2017; **99**(2): 169-77.

16. Soares MO, Welton NJ, Harrison DA, et al. Intravenous immunoglobulin for severe sepsis and septic shock: clinical effectiveness, cost-effectiveness and value of a further randomised controlled trial. *Crit Care* 2014; **18**(6): 649.

17. Wasserman RL. Clinical Practice Experience with HyQvia in Adults Using Alternative Dosing Regimens and Pediatric Patients: A Retrospective Study. *Advances in therapy* 2020; **37**(4): 1536-49.

18. Wiesik-Szewczyk E, Sołdacki D, Paczek L, Jahnz-Różyk K. Facilitated Subcutaneous Immunoglobulin Replacement Therapy in Clinical Practice: A Two Center, Long-Term Retrospective Observation in Adults With Primary Immunodeficiencies. *Front Immunol* 2020; **11**: 981.

19. Absoud M, Lim MJ. Intravenous immunoglobulin in paediatric neurology: evaluating effective usage and outcomes. *Developmental medicine and child neurology* 2016; **58**(11): 1105-6.

20. Eftimov F, Winer JB, Vermeulen M, de Haan R, van Schaik IN. Intravenous immunoglobulin for chronic inflammatory demyelinating polyradiculoneuropathy. *Cochrane Database Syst Rev* 2013; (12): CD001797.

21. Furlan JC, Barth D, Barnett C, Bril V. Cost-minimization analysis comparing intravenous immunoglobulin with plasma exchange in the management of patients with myasthenia gravis. *Muscle Nerve* 2016; **53**(6): 872-6.

22. Gelinas D, Katz J, Nisbet P, England JD. Current practice patterns in CIDP: A cross-sectional survey of neurologists in the United States. *J Neurol Sci* 2019; **397**: 84-91.

23. Hughes RAC, Donofrio P, Bril V, et al. Intravenous immune globulin (10% caprylate-chromatography purified) for the treatment of chronic inflammatory demyelinating polyradiculoneuropathy (ICE study): a randomised placebo-controlled trial. *The Lancet Neurology* 2008; **7**(2): 136-44.

24. Lazzaro C, Lopiano L, Cocito D. Subcutaneous vs intravenous administration of immunoglobulin in chronic inflammatory demyelinating polyneuropathy: an Italian cost-minimization analysis. *Neurol Sci* 2014; **35**(7): 1023-34.

25. Le Masson G, Sole G, Desnuelle C, et al. Home versus hospital immunoglobulin treatment for autoimmune neuropathies: A cost minimization analysis. *Brain Behav* 2018; **8**(2): e00923.

26. Maheshwari A, Sharma RR, Prinja S, et al. Cost-minimization analysis in the Indian subcontinent for treating Guillain Barre Syndrome patients with therapeutic plasma exchange as compared to intravenous immunoglobulin. *J Clin Apher* 2018; **33**(6): 631-7.

27. Meyer A, Scire CA, Talarico R, et al. Idiopathic inflammatory myopathies: narrative review of unmet needs in clinical practice guidelines. *RMD open* 2018; **4**(Suppl 1): e000784.

28. O'Connell K, Ramdas S, Palace J. Management of Juvenile Myasthenia Gravis. *Frontiers in neurology* 2020; **11**: 743.

29. Press R, Hiew FL, Rajabally YA. Steroids for chronic inflammatory demyelinating polyradiculoneuropathy: evidence base and clinical practice. *Acta Neurol Scand* 2016; **133**(4): 228-38.

30. Rajabally YA. Subcutaneous immunoglobulin therapy for inflammatory neuropathy: current evidence base and future prospects. *Journal of neurology, neurosurgery, and psychiatry* 2014; **85**(6): 631-7.

31. Sanders DB, Wolfe GI, Narayanaswami P. Developing treatment guidelines for myasthenia gravis. *Annals of the New York Academy of Sciences* 2018; **1412**(1): 95-101.

32. Stangel M, Gold R, Pittrow D, et al. Treatment of patients with multifocal motor neuropathy with immunoglobulins in clinical practice: the SIGNS registry. *Ther Adv Neurol Disord* 2016; **9**(3): 165-79.

33. Tsai CP. Pharmacoeconomics of intravenous immunoglobulin in various neurological disorders. *Acta Neurol Taiwan* 2010; **19**(4): 303-8.

34. van Schaik IN, Bril V, van Geloven N, et al. Subcutaneous immunoglobulin for maintenance treatment in chronic inflammatory demyelinating polyneuropathy (PATH): a randomised, double-blind, placebo-controlled, phase 3 trial. *The Lancet Neurology* 2018; **17**(1): 35-46.

35. Verboon C, Doets AY, Galassi G, et al. Current treatment practice of Guillain-Barré syndrome. *Neurology* 2019; **93**(1): e59-e76.

36. Winters JL, Brown D, Hazard E, Chainani A, Andrzejewski C, Jr. Cost-minimization analysis of the direct costs of TPE and IVIg in the treatment of Guillain-Barre syndrome. *BMC health services research* 2011; **11**: 101.

37. Yokoyama K, Hattori N. Management of myasthenia gravis in daily practice for general neurologists and healthcare professionals. *Clinical and Experimental Neuroimmunology* 2017; **8**(2): 162-70.

38. Sung N, Han AR, Park CW, et al. Intravenous immunoglobulin G in women with reproductive failure: The Korean Society for Reproductive Immunology practice guidelines. *Clin Exp Reprod Med* 2017; **44**(1): 1-7.

39. Shaulov T, Sierra S, Sylvestre C. Recurrent implantation failure in IVF: A Canadian Fertility and Andrology Society Clinical Practice Guideline. *Reprod Biomed Online* 2020; **41**(5): 819-33.

40. Callec R, Unlu O, Olivier M, Denis W, Stephane Z. Effectiveness of treatment in the secondary prevention of obstetric complications in the antiphospholipid syndrome: Systematic review and meta-analysis. *Human Reproduction* 2016; **Conference**(Supplement 1): i31.

41. Okubo Y, Michihata N, Morisaki N, et al. Recent trends in practice patterns and comparisons between immunoglobulin and corticosteroid in pediatric immune thrombocytopenia. *Int J Hematol* 2018; **107**(1): 75-82.

42. Wegnelius G, Bremme K, Lindqvist PG. Efficacy of treatment immune thrombocytopenic purpura in pregnancy with corticosteroids and intravenous immunoglobulin: a prospective follow-up of suggested practice. *Blood Coagul Fibrinolysis* 2018; **29**(2): 141-7.

43. Colla L, Diena D, Rossetti M, et al. Immunosuppression in pregnant women with renal disease: review of the latest evidence in the biologics era. *J Nephrol* 2018; **31**(3): 361-83.

44. Barut K, Sahin S, Adrovic A, Kasapcopur O. Diagnostic approach and current treatment options in childhood vasculitis. *Türk Pediatri Arşivi* 2015; **50**(4): 194-205.

45. Lieberman L, Greinacher A, Murphy MF, et al. Fetal and neonatal alloimmune thrombocytopenia: recommendations for evidence-based practice, an international approach. *Br J Haematol* 2019.

46. Prefumo F, Fichera A, Fratelli N, Sartori E. Fetal anemia: Diagnosis and management. *Best practice & research Clinical obstetrics & gynaecology* 2019.

47. Shehata N, Palda V, Bowen T, et al. The use of immunoglobulin therapy for patients with primary immune deficiency: an evidence-based practice guideline. *Transfus Med Rev* 2010; **24 Suppl 1**: S28-50.

48. Vultaggio A, Azzari C, Milito C, et al. Subcutaneous immunoglobulin replacement therapy in patients with primary immunodeficiency in routine clinical practice: the VISPO prospective multicenter study. *Clin Drug Investig* 2015; **35**(3): 179-85.

49. Iro MA, Martin NG, Absoud M, Pollard AJ. Intravenous immunoglobulin for the treatment of childhood encephalitis. *Cochrane Database Syst Rev* 2017; **10**: CD011367.

50. Gadian J, Kirk E, Holliday K, Lim M, Absoud M. Systematic review of immunoglobulin use in paediatric neurological and neurodevelopmental disorders. *Dev Med Child Neurol* 2017; **59**(2): 136-44.

51. Hopkins SE. Acute Flaccid Myelitis: Etiologic Challenges, Diagnostic and Management Considerations. *Curr Treat Options Neurol* 2017; **19**(12): 48.

52. Nosadini M, Mohammad SS, Suppiej A, Sartori S, Dale RC. Intravenous immunoglobulin in paediatric neurology: safety, adherence to guidelines, and long-term outcome. *Dev Med Child Neurol* 2016; **58**(11): 1180-92.

53. Rodriguez MM, Wagner-Weiner L. Intravenous Immunoglobulin in Pediatric Rheumatology: When to Use It and What Is the Evidence. *Pediatr Ann* 2017; **46**(1): e19-e24.

54. Florescu DF, Schaenman JM. Adenovirus in solid organ transplant recipients: Guidelines from the American Society of Transplantation Infectious Diseases Community of Practice. *Clinical transplantation* 2019; **33**(9): e13527.

55. Shehata N, Palda VA, Meyer RM, et al. The use of immunoglobulin therapy for patients undergoing solid organ transplantation: an evidence-based practice guideline. *Transfus Med Rev* 2010; **24 Suppl 1**: S7-s27.

56. Barmettler S, Ong MS, Farmer JR, Choi H, Walter J. Association of Immunoglobulin Levels, Infectious Risk, and Mortality With Rituximab and Hypogammaglobulinemia. *JAMA Netw Open* 2018; **1**(7): e184169.

57. Bhella S, Majhail NS, Betcher J, et al. Choosing Wisely BMT: American Society for Blood and Marrow Transplantation and Canadian Blood and Marrow Transplant Group's List of 5 Tests and Treatments to Question in Blood and Marrow Transplantation. *Biol Blood Marrow Transplant* 2018; **24**(5): 909-13.

58. Brand A, De Angelis V, Vuk T, Garraud O, Lozano M, Politis D. Review of indications for immunoglobulin (IG) use: Narrowing the gap between supply and demand. *Transfusion clinique et biologique : journal de la Societe francaise de transfusion sanguine* 2021; **28**(1): 96-122.

59. Calizzani G, Vaglio S, Candura F, et al. The evolution of the regulatory framework for the plasma and plasma-derived medicinal products system in Italy. *Blood transfusion = Trasfusione del sangue* 2013; **11 Suppl 4**: s6-12.

60. De Angelis V, Breda A. Plasma-derived medicinal products self-sufficiency from national plasma: to what extent? *Blood transfusion = Trasfusione del sangue* 2013; **11 Suppl 4**: s132-7.

61. Farrugia A, Cassar J. Plasma-derived medicines: access and usage issues. *Blood transfusion = Trasfusione del sangue* 2012; **10**(3): 273-8.

62. Farrugia A. Are we optimising outcomes in Australia's framework for the supply of plasma-derived medicines? *Med J Aust* 2016; **205**(7): 336.

63. Grazzini G, Ceccarelli A, Calteri D, Catalano L, Calizzani G, Cicchetti A. Sustainability of a public system for plasma collection, contract fractionation and plasma-derived medicinal product manufacturing. *Blood transfusion = Trasfusione del sangue* 2013; **11 Suppl 4**: s138-47.

64. Kohn DB, Kuo CY. New frontiers in the therapy of primary immunodeficiency: From gene addition to gene editing. *J Allergy Clin Immunol* 2017; **139**(3): 726-32.

65. Lanzoni M, Biffoli C, Candura F, Calizzani G, Vaglio S, Grazzini G. Plasma-derived medicinal products in Italy: information sources and flows. *Blood transfusion = Trasfusione del sangue* 2013; **11 Suppl 4**: s13-7.

66. Lanzoni M, Candura F, Calizzani G, Biffoli C, Grazzini G. Public expenditure for plasma-derived and recombinant medicinal products in Italy. *Blood transfusion = Trasfusione del sangue* 2013; **11 Suppl 4**: s110-7.

67. Murphy MSQ, Tinmouth A, Goldman M, et al. Trends in IVIG use at a tertiary care Canadian center and impact of provincial use mitigation strategies: 10-year retrospective study with interrupted time series analysis. *Transfusion* 2019.

68. O'Mahony B. The Dublin Consensus Statement 2012 on optimised supply of plasma-derived medicinal products. *Blood transfusion = Trasfusione del sangue* 2013; **11**(4): 623-6.

69. Perez EE, Orange JS, Bonilla F, et al. Update on the use of immunoglobulin in human disease: A review of evidence. *J Allergy Clin Immunol* 2017; **139**(3S): S1-S46.

70. Shapiro RS, Borte M. 7th International Immunoglobulin Conference: Immunoglobulin in clinical practice. *Clin Exp Immunol* 2014; **178 Suppl 1**: 86.

71. Strengers PF, Klein HG. Plasma is a strategic resource. *Transfusion* 2016; **56**(12): 3133-7.

72. Strengers PFW. Evidence-based clinical indications of plasma products and future prospects. *Annals of Blood* 2017; **2**: 20.

73. Watad A, Amital H, Shoenfeld Y. Intravenous immunoglobulin: a biological corticosteroid-sparing agent in some autoimmune conditions. *Lupus* 2017.

74. Blau IW, Conlon N, Petermann R, Nikolov N, Plesner T. Facilitated subcutaneous immunoglobulin administration (fSCIg): a new treatment option for patients with secondary immune deficiencies. *Expert Rev Clin Immunol* 2016; **12**(7): 705-11.

75. Grazzini G, Mannucci PM, Oleari F. Plasma-derived medicinal products: demand and clinical use. *Blood transfusion = Trasfusione del sangue* 2013; **11 Suppl 4**: s2-5.

76. Stonebraker JS, Farrugia A, Gathmann B, Orange JS. Modeling Primary Immunodeficiency Disease Epidemiology and Its Treatment to Estimate Latent Therapeutic Demand for Immunoglobulin. *Journal of Clinical Immunology* 2014; **34**(2): 233-44.

77. Stonebraker JS, Hajjar J, Orange JS. Latent therapeutic demand model for the immunoglobulin replacement therapy of primary immune deficiency disorders in the USA. *Vox Sang* 2018.

78. Farrugia A, Bansal M, Marjanovic I. Estimation of the latent therapeutic demand for immunoglobulin therapies in autoimmune neuropathies in the United States. *Vox Sang* 2021.

79. Cheraghali AM, Abolghasemi H. Improving availability and affordability of plasma-derived medicines. *Biologicals* 2010; **38**(1): 81-6.

80. Farrugia A, Evers T, Falcou PF, Burnouf T, Amorim L, Thomas S. Plasma fractionation issues. *Biologicals* 2009; **37**(2): 88-93.

81. Flanagan P. Self-sufficiency in plasma supply. *Vox Sanguinis* 2017; **Conference**.

82. Godin G, Germain M. Predicting first lifetime plasma donation among whole blood donors. *Transfusion* 2013; **53 Suppl 5**: 157S-61S.

83. Rautonen J. Self-sufficiency, free trade and safety. *Biologicals* 2010; **38**(1): 97-9.

84. Späth PJ, Schneider C, von GS. Clinical Use and Therapeutic Potential of IVIG/SCIG, Plasma-Derived IgA or IgM, and Other Alternative Immunoglobulin Preparations. *Arch Immunol Ther Exp (Warsz )* 2017; **65**(3): 215-31.

85. Veldhuizen I, van DA. Motivational differences between whole blood and plasma donors already exist before their first donation experience. *Transfusion* 2013; **53**(8): 1678-86.

86. HealthCanada. Protecting Access to Immune Globulins for Canadians:

Final Report of the Expert Panel on Immune Globulin Product Supply and Related Impacts in Canada, 2018.

87. Ducruet T, Levasseur MC, Des Roches A, Kafal A, Dicaire R, Haddad E. Pharmacoeconomic advantages of subcutaneous versus intravenous immunoglobulin treatment in a Canadian pediatric center. *J Allergy Clin Immunol* 2013; **131**(2): 585-7.e1-3.

88. Grabowski HG, Manning RL. An Economic Analysis of Global Policy Proposals to Prohibit Compensation of Blood Plasma Donors. *International Journal of the Economics of Business* 2016; **23**(2): 149-66.

89. Perraudin C, Bourdin A, Vicino A, Kuntzer T, Bugnon O, Berger J. Home-based subcutaneous immunoglobulin for chronic inflammatory demyelinating polyneuropathy patients: A Swiss cost-minimization analysis. *PloS one* 2020; **15**(11): e0242630.

90. Petrini C. Production of plasma-derived medicinal products: ethical implications for blood donation and donors. *Blood transfusion = Trasfusione del sangue* 2014; **12 Suppl 1**: s389-94.

91. Prevot J, Jolles S. Global immunoglobulin supply: steaming towards the iceberg? *Current opinion in allergy and clinical immunology* 2020; **20**(6): 557-64.

92. Shapiro RS, Boyle M. Payor issues: barriers to optimal management of patients with primary immunodeficiency. *Journal of clinical immunology* 2012; **32 Suppl 2**: S410-4.

93. Windegger TM, Nghiem S, Nguyen KH, Fung YL, Scuffham PA. Cost-utility analysis comparing hospital-based intravenous immunoglobulin with home-based subcutaneous immunoglobulin in patients with secondary immunodeficiency. *Vox Sang* 2019; **114**(3): 237-46.

94. Derman BA, Schlei Z, Parsad S, Mullane K, Knoebel RW. Changes in Intravenous Immunoglobulin Usage for Hypogammaglobulinemia After Implementation of a Stewardship Program. *JCO oncology practice* 2021; **17**(3): e445-e53.

95. Edington HJ, Sutton KS, Bennett C, Chandrakasan S, Sterner-Allison J, Castellino SM. Dealing with a critical national shortage-Approaches to triaging immune globulin supply in pediatric hematology and oncology. *Pediatr Blood Cancer* 2020; **67**(7): e28260.

96. Jones TR, Wilkie G. First steps into lean at the Australian red cross blood service. *Vox Sanguinis* 2016; **Conference**(Supplement 2): 14.

97. Tsapepas D, Der-Nigoghossian C, Patel K, Berger K, Vawdrey DK, Salmasian H. Medication stewardship using computerized clinical decision support: A case study on intravenous immunoglobulins. *Pharmacology research & perspectives* 2019; **7**(5): e00508.
