# Supplementary material for: Using a scenario approach to assess for the current and future demand of immunoglobulins: An interview and literature study from The Netherlands
Source: Transfus Med. 2022 Jun 24;32(5):410–21. doi: 10.1111/tme.12889 (PMC9795925; doi:10.1111/tme.12889)
Supplement: Supplementary file 2 — Supplementary Table 2 Factors that could increase Ig demand Supplementary Table 3: Factors that could decrease Ig demand. Supplementary Table 4: Miscellaneous but important factors [file TME-32-410-s001.docx]

**Supplementary Information**

Supplementary Tables 2-4 provide comparative descriptions regarding transformational factors and its effects from the scoping review and interviews.

**Supplementary Table 2: Factors that could increase Ig demand**

|  | **From literature** | **From interviews** |
| --- | --- | --- |
| Social factors | - Aging population who become diseased and need treatments (especially for secondary care) and - Increasing weight (as IVIG is weight-based) - Older age of pregnant women - Growth of population with chronic diseases | - Growing aging population who become diseased and need treatments - No age limits for stem cell transplantations - Increasing physician awareness to diagnose patients and prescribe Ig through education, diagnostic tools, or using algorithms embedded in electronic patient record systems - Increasing physician awareness through experience and word of mouth: physicians who used Ig beneficially for patients and mentoring other physicians to do the same |
| Technological/clinical | - Increased secondary immunodeficiency (from increased immune modulating and cellular therapies) - Consequences of CAR-T cell therapy, which may cause prolonged hypogammaglobulinaemia - Consequences of Rituximab therapy - Improved diagnostics for PID | - If oncology protocols all harmonized with using Ig suppletion after oncological treatments - Using an algorithm to identify potential patients in medical records - Improved (a-fucosylated) IG - Consequences of anti Fc-Rn antibodies therapy - Increasing complications or consequences from other immunosuppressive treatments, including stem cell transplantations, cellular therapies, new drugs which need IG as secondary support - Pediatric trial (IPAD) in Netherlands to assess if increased Ig dosage will prevent pulmonary diseases and lead to improved outcomes in   patients with PIDs |
| Legal |  | - EMA Guidelines 2019: revisions to the Summary of Product Characteristics (SmPC) which now include CIDP, MMN, and broader definition for secondary immune deficiencies |

**Supplementary Table 3: Factors that could decrease Ig demand**

|  | **From literature** | **From interviews** |
| --- | --- | --- |
| Social factors |  | - Negative public perceptions due to a blood scandal or negative perception of medicines from human origins |
| Technological/clinical factors | - New products for rheumatologic diseases and increased use of thrombopoietin agonists for immune thrombocytopenia (mentioned in Discussion) - Use of steroids as first-line treatment option in myasthenia gravis - Rituximab therapy, as replacement for IVIG - Lack of sufficient RCTs that prove IVIG’s efficacy | - Recombinant products - Alternatives to IVIG, such as biological products, targeted therapies like monoclonal drugs, neonatal receptor (Fc-Rn receptor) blocker that has the same effect as IVIG (blocks the Fc-Rn receptor) but with mechanism differences, or Immunoglobulin G-degrading enzyme of Streptococcus pyogenes (IdeS, a cysteine protease that cleaves IgG antibodies into fragments) - Gene-correcting therapies (gene therapy, stem cell transplantation, CRISPR-Cas) for certain primary immune deficiencies with a monogenetic cause of the disease - Using Rituximab therapy in CIDP patients (trial undergoing) - Studies that explain IVIG’s mechanism to create alternatives to it - RCTs that disprove IVIG’s efficacy in various diseases; specifically, neurology trials for stopping/tapering patients off IVIG (e.g in CIDP) |
| Ecological |  | - Infectious disease transmitted through plasma donations, not detectable through screening |
| Economic/political/legal | - High cost of IVIG | - High cost of IVIG - Increased focus on using cost-effectiveness in choosing products - Dutch Transfer Act 2021, with the aim of controlling Ig usage - Strictness with off-label IVIG usage - Ethical stance not to harm plasma donors in any way (including for remunerated donations) |

**Supplementary Table 4: Miscellaneous but important factors**

|  | **From literature** | **From interviews** |
| --- | --- | --- |
| Societal factors | - Supply issues, interrelated to logistical factors of contract fractionation and recruiting/retaining plasma donors | - Freedom of choice: having branding and application options to choose from - Increasing usage of SCIg or facilitated SCIg (fSCIg) - Sufficient plasma donors - Marketing/organizational strategies to obtain donors - (Non)-remuneration of donors - Ensuring the (positive) reputation of organization |
| Technological factors | - Optimized use of plasma-derived proteins | - Production factors: Technological changes to have greater yield in the same pool of plasma |
| Economic/political/ legal factors | - Using subcutaneous immunoglobulins (SCIg) could be substantially cost-saving for multiple parties involved^31,62,63^ although the impact on demand is yet unknown due to dosing differences - Supply issues, interrelated to factors of contract fractionation, access issues, donor remuneration, and self-sufficiency - Reimbursement issues, related to the pricing of products, and barriers from patients and insurance companies | - Price-setting of Ig products - Market sizes of different countries for Ig products (and these products being diverted to the more lucrative markets) - Dependency on U.S. plasma - Specific governmental regulations - Competition within the pharmaceutical industry and consequential supply issues - Substantial power of commercial companies - High rate of off-label usage |

| **((**("Pharmaceutical Preparations/economics"[Mesh] OR "Pharmaceutical Preparations/supply and distribution"[Mesh] OR "Immunologic Factors/economics"[Mesh] OR "Immunologic Factors/supply and distribution"[Mesh] OR "Immunoglobulins, Intravenous/economics"[majr] OR "Legislation, Drug"[Mesh:NoExp]) AND ("plasma-derived"[tw] OR "plasma derived medical products"[tw] OR "plasma derived medicinal products"[tw] OR "plasma derived medicines"[tw] OR "plasma derived preparations"[tw] OR "plasma derived product"[tw] OR "plasma derived products"[tw] OR "plasma derived drug"[tw] OR "plasma derived components"[tw] OR "plasma derived concentrate"[tw] OR "plasma derived concentrates"[tw] OR "plasma derived biological medicines"[tw] OR "plasma derived blood products"[tw] OR "plasma derived protein"[tw] OR "plasma derived proteins"[tw] OR "plasma derived therapeutic products"[tw] OR "plasma derived therapeutic proteins"[tw] OR "plasma derived therapeutics"[tw] OR "plasma derived therapies"[tw] OR "plasma derived drugs" [tw] OR (("PDMP"[tw] OR "PDMPs"[tw]) AND "plasma"[tw]) OR "Plasma Products"[tw] OR "Plasma Product"[tw] OR **(("Pharmaceutical Preparations"[mesh] OR "Biological Products"[mesh] OR "Biopharmaceutics"[mesh]) AND ("Plasma"[mesh] OR "plasma"[ti]))** OR "Immunoglobulins, Intravenous"[Mesh] OR "Intravenous Immunoglobulin"[tw] OR "Intravenous Immunoglobulins"[tw] OR "Intravenous IG"[tw] OR "IVIG"[tw] OR "IVIGs"[tw] OR "Intravenous Immune Globulin"[tw] OR "IV Immunoglobulins"[tw] OR "Flebogamma DIF"[tw] OR "Gamunex"[tw] OR "Globulin-N"[tw] OR "Globulin N"[tw] OR "Intraglobin"[tw] OR "Intraglobin F"[tw] OR "Gammagard"[tw] OR "Gamimune"[tw] OR "Gamimmune"[tw] OR "Modified Immune Globulin"[tw] OR "Privigen"[tw] OR "Sandoglobulin"[tw] OR "Venoglobulin"[tw] OR "Venoglobulin-I"[tw] OR "Venoglobulin I"[tw] OR "Iveegam"[tw] OR "Alphaglobin"[tw] OR "Endobulin"[tw] OR "Gamimune N"[tw] OR "Gamimmune N"[tw] OR "Gammonativ"[tw])**) OR (("current practice"[tw] OR "current clinical practice"[tw] OR "practice"[ti] OR "usage"[ti] OR "daily practice"[tw] OR "current usage"[tw] OR "current use"[tw] OR "common practice"[tw])** AND ("plasma-derived"[tw] OR "plasma derived medical products"[tw] OR "plasma derived medicinal products"[tw] OR "plasma derived medicines"[tw] OR "plasma derived preparations"[tw] OR "plasma derived product"[tw] OR "plasma derived products"[tw] OR "plasma derived drug"[tw] OR "plasma derived components"[tw] OR "plasma derived concentrate"[tw] OR "plasma derived concentrates"[tw] OR "plasma derived biological medicines"[tw] OR "plasma derived blood products"[tw] OR "plasma derived protein"[tw] OR "plasma derived proteins"[tw] OR "plasma derived therapeutic products"[tw] OR "plasma derived therapeutic proteins"[tw] OR "plasma derived therapeutics"[tw] OR "plasma derived therapies"[tw] OR "plasma derived drugs" [tw] OR (("PDMP"[tw] OR "PDMPs"[tw]) AND "plasma"[tw]) OR "Plasma Products"[tw] OR "Plasma Product"[tw] OR **(("Pharmaceutical Preparations"[mesh] OR "Biological Products"[mesh] OR "Biopharmaceutics"[mesh]) AND ("Plasma"[mesh] OR "plasma"[ti]))** OR "Immunoglobulins, Intravenous"[Mesh] OR "Intravenous Immunoglobulin"[tw] OR "Intravenous Immunoglobulins"[tw] OR "Intravenous IG"[tw] OR "IVIG"[tw] OR "IVIGs"[tw] OR "Intravenous Immune Globulin"[tw] OR "IV Immunoglobulins"[tw] OR "Flebogamma DIF"[tw] OR "Gamunex"[tw] OR "Globulin-N"[tw] OR "Globulin N"[tw] OR "Intraglobin"[tw] OR "Intraglobin F"[tw] OR "Gammagard"[tw] OR "Gamimune"[tw] OR "Gamimmune"[tw] OR "Modified Immune Globulin"[tw] OR "Privigen"[tw] OR "Sandoglobulin"[tw] OR "Venoglobulin"[tw] OR "Venoglobulin-I"[tw] OR "Venoglobulin I"[tw] OR "Iveegam"[tw] OR "Alphaglobin"[tw] OR "Endobulin"[tw] OR "Gamimune N"[tw] OR "Gamimmune N"[tw] OR "Gammonativ"[tw])**) OR (**((demand*[ti] NOT "on-demand"[ti]) OR "demands"[ti] OR "supply"[ti] OR "supplies"[ti] OR "supplied"[ti] OR "future demand"[tw] OR "future demands"[tw] OR "demand change"[tw] OR "demand changes"[tw] OR "demand characteristics"[tw] OR "demand prediction"[tw] OR "future supplies"[tw] OR "future supply"[tw] OR "supply and distribution"[subheading] OR "Resource Allocation"[mesh] OR (("demand"[tw] OR "demands"[tw]) AND ("future"[tw] OR predict*[tw] OR "change"[tw] OR "changes"[tw] OR "changing"[tw]))) AND ("plasma-derived"[tw] OR "plasma derived medical products"[tw] OR "plasma derived medicinal products"[tw] OR "plasma derived medicines"[tw] OR "plasma derived preparations"[tw] OR "plasma derived product"[tw] OR "plasma derived products"[tw] OR "plasma derived drug"[tw] OR "plasma derived components"[tw] OR "plasma derived concentrate"[tw] OR "plasma derived concentrates"[tw] OR "plasma derived biological medicines"[tw] OR "plasma derived blood products"[tw] OR "plasma derived protein"[tw] OR "plasma derived proteins"[tw] OR "plasma derived therapeutic products"[tw] OR "plasma derived therapeutic proteins"[tw] OR "plasma derived therapeutics"[tw] OR "plasma derived therapies"[tw] OR "plasma derived drugs" [tw] OR (("PDMP"[tw] OR "PDMPs"[tw]) AND "plasma"[tw]) OR "Plasma Products"[tw] OR "Plasma Product"[tw] OR **(("Pharmaceutical Preparations"[mesh] OR "Biological Products"[mesh] OR "Biopharmaceutics"[mesh]) AND ("Plasma"[mesh] OR "plasma"[ti]))** OR "Immunoglobulins, Intravenous"[Mesh] OR "Intravenous Immunoglobulin"[tw] OR "Intravenous Immunoglobulins"[tw] OR "Intravenous IG"[tw] OR "IVIG"[tw] OR "IVIGs"[tw] OR "Intravenous Immune Globulin"[tw] OR "IV Immunoglobulins"[tw] OR "Flebogamma DIF"[tw] OR "Gamunex"[tw] OR "Globulin-N"[tw] OR "Globulin N"[tw] OR "Intraglobin"[tw] OR "Intraglobin F"[tw] OR "Gammagard"[tw] OR "Gamimune"[tw] OR "Gamimmune"[tw] OR "Modified Immune Globulin"[tw] OR "Privigen"[tw] OR "Sandoglobulin"[tw] OR "Venoglobulin"[tw] OR "Venoglobulin-I"[tw] OR "Venoglobulin I"[tw] OR "Iveegam"[tw] OR "Alphaglobin"[tw] OR "Endobulin"[tw] OR "Gamimune N"[tw] OR "Gamimmune N"[tw] OR "Gammonativ"[tw])**))** NOT ("Animals"[mesh] NOT "Humans"[mesh]) AND ("2008/01/01"[PDAT] : "3000/12/31"[PDAT])  **Appendix A: Three-part scoping review search strategy and exclusion criteria** |
| --- |
| Exclusion criteria:   - No animals - No studies before 2010 - No studies in a language other than English or Dutch - No studies regarding ‘on-label’ or ‘established’ indications with no reported changes (e.g. dosage) that would warrant impact on future demand - No full text available |

**Appendix B – Interview guide**

1. Introductions
   1. Myself and the purpose of this research
   2. What is the respondent´s role and function?
   3. How long has the respondent been in this role and/or involved in the plasma industry?
2. Present
   1. (For clinicians) How many patients are treated with IVIG? What diseases/disorders do these patients have? How much IVIG is used for their treatments?
   2. (For non-clinicians) Has the respondent noticed any trends regarding IVIG demand?
   3. What are key transformational factors that would impact IVIG demand and *to what effect*?
      1. Social
      2. Technological
      3. Economical
      4. Ecological
      5. Political
      6. Legal
3. Future
   1. What are potential new areas of medical need that could be explored?
   2. What are potential drivers for IVIG demand?
   3. What is the expected change in demand for IVIG in 10 years?
   4. What is the expected change in demand for IVIG in 15 years?
   5. What are the organizational implications for Sanquin’s Blood Bank or Sanquin’s Plasma Products (i.e. with regards to specific requirements of products, infrastructure, facilities, and staffing) ?
4. Conclusion
   1. Clarification from both sides
   2. Networking
   3. Thanks/assurance of anonymity
